# Room-temperature multiferroicity and diversified magnetoelectric couplings in 2D materials

**DOI:** 10.1093/nsr/nwz169

**Published:** 2019-11-05

**Authors:** Tingting Zhong, Xiaoyong Li, Menghao Wu, Jun-Ming Liu

**Affiliations:** 1 School of Physics and Wuhan National High Magnetic Field Center, Huazhong University of Science and Technology, Wuhan 430074, China; 2 Laboratory of Solid State Microstructures, Nanjing University, Nanjing 210093, China

**Keywords:** 2D multiferroics, room-temperature ferromagnetism and ferroelectricity, diversified magnetoelectric couplings, vertical polarizations, first-principles calculations

## Abstract

Multiferroics are rare in nature due to the mutual exclusive origins of magnetism and ferroelectricity. The simultaneous coexistence of robust magnetism/ferroelectricity and strong magnetoelectric coupling in single multiferroics is hitherto unreported, which may also be attributed to their potential conflictions. In this paper, we show the first-principles evidence of such desired coexistence in ultrathin-layer CuCrS_2_ and CuCrSe_2_. The vertical ferroelectricity is neither induced by an empty d shell nor spin-driven, giving rise to an alternative possibility of resolving those intrinsic exclusions and contradictions. Compared with their bulk phases, the ferromagnetism in the thin-layer structures (two–six layers) can be greatly stabilized due to the enhanced carrier density and orbital shifting by vertical polarization, and the Curie temperatures of both ferromagnetism and ferroelectricity can be above room temperature. Moreover, a considerable net magnetization can be reversed upon ferroelectric switching, where the change in spin-resolved band structure also renders efficient ‘magnetic reading + electrical writing’. The thickness-different layers may even exhibit diversified types of magnetoelectric coupling, which both enriches the physics of multiferroics and facilitates their practical applications.

## INTRODUCTION

The recent progress in 2D van der Waals ferroelectrics [1] may revolutionize the applications of ferroelectric (FE) materials in nanoelectronics. Their atomic thickness renders high-density integration and their clean van der Waals interfaces allow for lattice mismatch, facilitating the epitaxial growth on various substrates including silicon; meanwhile, many 2D materials are also high-mobility low-bandgap semiconductors. The designs of 2D ferroelectrics since 2013 can be classified into two types. Predictions of inducing FE in prevalent non-polar 2D materials have been reported in several studies [2–4], while their experimental realizations have been scarcely reported; in comparison, the first-principle predictions of intrinsic FE in several 2D materials have been experimentally confirmed, such as IV–VI group compound [5–8], In_2_Se_3_ [9,10], van der Waals bilayer [11–13], and Bi_2_O_2_Se [14,15], where CuInP_2_S_6_ is currently the only confirmed 2D FE material without a related advance prediction [16,17].

2D multiferroics have also been explored [18–24], where magnetoelectric (ME) couplings may be more prevalent and diversified compared with traditional multiferroics, which are highly desirable for realizing efficient ‘magnetic reading + electric writing’. Multiferroic materials that are both FE and magnetic are rare in nature due to the mutual exclusive origins of the two orders (empty d shell for conventional FE order and partially filled d shell for magnetic order). Meanwhile, for traditional multiferroics, the ME coupling is weak in type-I multiferroics with ferroelectricity and magnetism arising respectively from different mechanisms, while for type-II multiferroics, where ferroelectricity is induced by magnetic order, their spin-driven FE polarizations (mostly < 0.01 C/m^2^) and Curie temperature (mostly < 150 K) are far below the values for practical applications [25]. It is the same case for previous designs of 2D type-I [2] and type-II multiferroics [26,27]. Recently, a new type of magnetoelectric coupling where spin distribution is altered via FE switching has been predicted in several 2D functionalized multiferroics [19,20,28]. Their coexistence of ferroelectricity/ferromagnetism and strong ME coupling indicates that the mutual exclusion might be avoided, although it is still challenging to obtain room-temperature magnetism or reverse the magnetization electrically. Experiments on those 2D functionalized multiferroics are still lacking. It seems that 2D intrinsic multiferroics are more desirable for a higher chance of experimental realizations, which is also implied by the syntheses of 2D FE mentioned above.

Herein our research is focused on CuCrX_2_ (X = S or Se), which have sparked considerable interest owing to their high efficiency of thermoelectric conversion [29–32]. They undergo structural transition from the *C*m to the *R*3m space group when T goes above 37.5 K and 55 K respectively [33,34], in which the X–Cr–X layers are separated by non-magnetic layers of monovalent Cu atoms. Below the low transition temperature (*C*m phase), they were revealed to be both antiferromagnetic (AFM) with weak spin-driven ferroelectricity (type-II) in previous reports [34,35], which are not feasible for practical applications like other type-II multiferroics. However, we note the centro-symmetry breaking by the considerable displacements of Cu ions at the *R*3m phase above the transition temperature, which may give rise to a much stronger ferroelectricity that is neither spin-driven nor induced by the empty d shell (the Cu^+^ ions should be with a closed d shell and prefer tetrahedral coordination). In this paper, we show the first-principles evidence for a new type of multiferroicity in a thin film of room-temperature *R*3m phase CuCrX_2_, which can be room-temperature robust with strong ME coupling for reversing a considerable magnetization electrically. Their ferromagnetism is stabilized by the enhanced carrier density and the vertical polarization-driven orbital shifting, while the formation of vertical 2D FE polarization can be attributed to the tetrahedral coordination of Cu ions. Also distinct from bulk systems, the vertical polarization is not diminished by the in-plane metallicity with enhanced carrier density because the electrons are vertically confined, which has been verified by the observed ferroelectric switching of metallic bilayer WTe_2_ [12,13]. Theoretically, the mutual exclusion between ferroelectricity and magnetism or between high Curie temperature and strong magnetoelectricity in traditional multiferroics can be avoided here.

## RESULTS AND DISCUSSION

### Bulk properties

**Figure 1.**
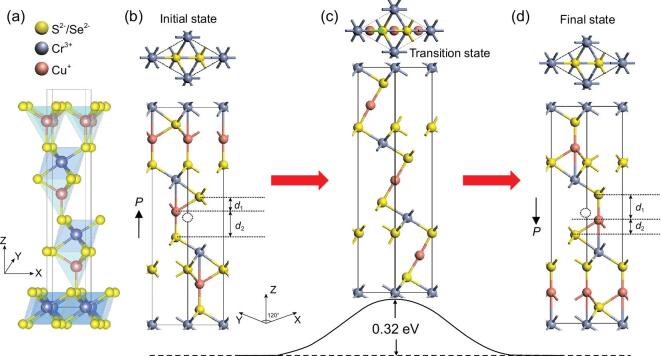
(a) The geometric structure of bulk CuCrX_2_. (b) The lattice structure of one FE state as the initial state. Here, the spontaneous polarization is generated via the shifting of a Cu^+^ ion from its high-symmetry (paraelectric) position (denoted by the open dashed dot). Non-zero upward polarization *P* can be expressed by distance *d*_2_ > *d*_1_ and it becomes zero if *d*_2_ = *d*_1_, where the polarization directions are marked by black arrows. (c) The transition state via which the FE switching pathway of CuCrS_2_ goes, upon the displacements of Cu ions tracked by the NEB method; this state is most likely the paraelectric phase. (d) The final FE state after the polarization switching where the downward polarization is expressed by distance *d*_2_ < *d*_1_.

We first check the electronic structure and multiferroic properties of bulk CuCrX_2_ (X = S or Se) lattices. The geometric structure of bulk CuCrX_2_ (*R*3m phase at ambient conditions) is displayed in Fig. 1a, which can be deemed as CrX_2_ layers intercalated by Cu atoms. According to the crystal field theory, sp^3^ tetrahedral bonding is generally favorable in energy for Cu^+^ with d^10^ electron configuration. As a result, each copper atom is tetrahedrally coordinated with one S/Se atom in one of the adjacent layers and three S/Se atoms of the other layer, so the vertical distances between Cu ions and two adjacent layers, *d*_1_ and *d*_2_, are different, generating the electric polarization. For the case of CuCrS_2_, *d*_2_ = 2.17 Å is much longer than *d*_1_ = 1.25 Å, as marked in Fig. 1b, contributing a vertical (upward) FE polarization. An equivalent state with reversed vertical polarization can be obtained upon the displacement of Cu ions, as shown in Fig. 1d. The estimated polarizations for CuCrS_2_ and CuCrSe_2_ are both around 0.19 C/m^2^, which will be switchable if there is a pathway to transform between those two states with a moderate barrier. Given such a pathway, both systems can be well-defined ferroelectrics.

As a paradigm example, we track the switching pathway for bulk CuCrS_2_ lattice by using the nudged elastic band (NEB) method, as displayed in Fig. 1b–d, noting here that the difference between *d*_1_ and *d*_2_ is enlarged just for clarification. It is seen that Cu ions will be bi-coordinated in the transition state (Fig. 1c, i.e. the paraelectric phase), where the switching barrier will be 0.32 eV per Cu ion. Such a barrier implies that a high electric field is likely to be required for the polarization reversal. Given such a barrier, one expects that a high voltage of 100 V will be required in order to generate a vertical electric field of 1 V/nm for a 100-nm-thick layer, which is too high for any practical applications. Therefore, one needs to search for an alternative approach via which such a barrier can be reduced remarkably.

For the magnetism, our results indicate an antiferromagnetic ground state with a magnetic moment of 2.84 μ_B_ per Cr ion for such a bulk lattice, suggesting that CuCrX_2_ is multiferroic. As shown in Fig. S1, the ground state is only 14.7 meV/f.u. lower in energy compared with the FM state, revealing a rather low Néel temperature. Such weak magnetism of Cr ions should be independent of the FE induced by the displacement of Cu ions, so herein magnetoelectric coupling will be a luxury. The Cr ions in each layer of bulk CuCrX_2_ are equivalent due to the translational symmetry, and each Cu ion possesses a filled 3d shell with a charge state of +1. The partial density of states (PDOS) results shown in Fig. S1 reveal a negligible hybridization between S and Cu near the Fermi level.

### Multiferroicity of ultrathin layers

**Figure 2.**
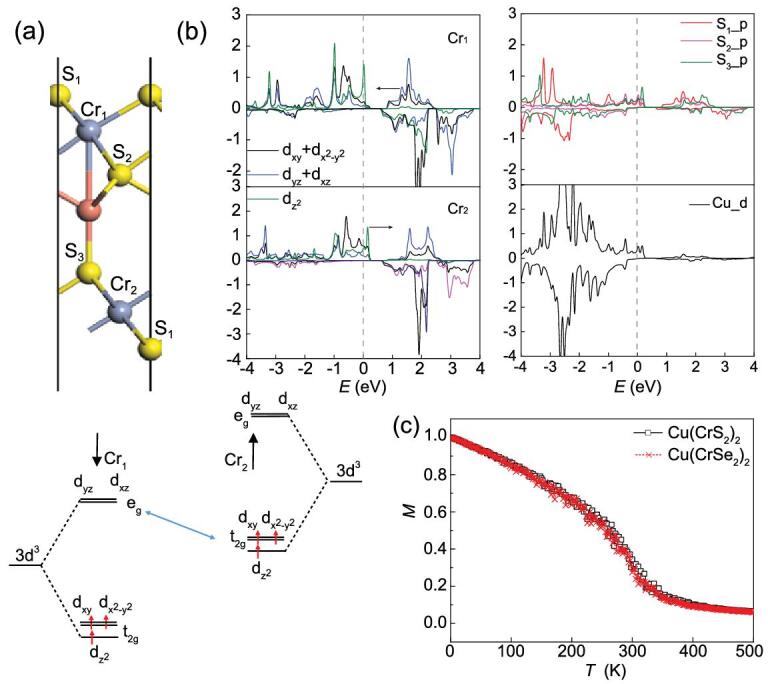
(a) Orbital analysis and (b) PDOS analysis of Cu(CrS_2_)_2_, where the vertical polarization breaks the orbital energy degeneracy of Cr_1_ and Cr_2_, and black arrows denote the shift of orbitals due to vertical polarization. (c) Monte Carlo simulated temperature dependence of magnetization of Cu(CrS_2_)_2_ and Cu(CrSe_2_)_2_.

Now we come to the situation of the ultrathin structure of CuCrX_2_. The situation becomes very different. For the thinnest structure composed of two CrX_2_ layers intercalated by Cu atoms, denoted as Cu(CrX_2_)_2_, as displayed in Fig. 2a, the lattice will be hole-doped due to the change of stoichiometric ratio (with a hole density of 8.6 × 10^14^ cm^−2^) and the hybridization can be greatly enhanced, as shown by the PDOS analysis in Fig. 2b. Such ultrathin layers might be fabricated by epitaxial growth, or electrochemical control of copper intercalation into CrX_2_ layers, which have been applied in the previous synthesis of copper-intercalated Bi_2_Se_3_ [36]. The band structure and PDOS of bulk system is presented in Fig. S1 as a reference. It is seen that the bulk lattice is gapped with 0.40 eV and favored with the antiferromagnetic ground state.

Unlike the bulk lattice, here for 2D Cu(CrX_2_)_2_, our calculations indicate that the interlayer and intralayer couplings are both ferromagnetic mainly due to much enhanced carrier density at the Fermi level majorly distributed by Cr_1_, which gives rise to in-plane metallicity. Another reason for the ferromagnetism can be attributed to orbital shift induced by the vertical polarization. This ferromagnetism can be understood qualitatively from the viewpoint of electronic structure. Due to the octahedral crystal field, as shown in Fig. 2a, the Cr-d orbitals split into lower t_2g_ that are half occupied and higher empty e_g_ orbitals. The FM coupling between two Cr^3+^ ions can be greatly enhanced when the energy gap between the occupied t_2g_ of one Cr^3+^ ion and the empty e_g_ orbital of another is reduced. Here due to the breaking of translational symmetry and polarization along the vertical direction, the orbitals of Cr_1_ in the upper layer will be shifted downwards while the orbitals of Cr_2_ in the lower layer will be shifted upwards. This can also be revealed by the PDOS in blue shifting towards the Fermi level for Cr_1_ and the PDOS in green shifting over the Fermi level for Cr_2_ in Fig. 2b, and the reduced e_g_(Cr_1_)-t_2g_(Cr_2_) gap will strengthen FM coupling.

For the interesting ferromagnetism in this ultrathin structure, we can estimate the Curie temperature using Monte Carlo simulations based on the Heisenberg model, where the spin Hamiltonian can be written as:

(1) \begin{equation*} {\boldsymbol{\hat{H}}} = {\rm{\ }} - \mathop \sum \limits_{{\rm{\langle i}}{{\boldsymbol{j} \rangle}}} {\boldsymbol{J}}{{\boldsymbol{\vec{s}}}_{\boldsymbol{i}}}\cdot{{\boldsymbol{\vec{s}}}_{\boldsymbol{j}}}, \end{equation*}

where *J* is defined as the nearest neighboring exchange coupling parameter (positive values favor FM coupling), which is calculated from the energy difference between different spin configurations using the Heyed–Scuseria–Ernzerhof (HSE) functional. As listed in Table S1, the value of intralayer *J*_1_ is significantly larger than interlayer *J*_2_. In our Monte Carlo simulations, a 2D 30 × 30 supercell is adopted, 2 × 10^5^ iterations at each temperature are employed and spins on all magnetic sites flip randomly. The magnetizations as a function of *T* for Cu(CrS_2_)_2_ and Cu(CrSe_2_)_2_ are simulated in Fig. 2c, both revealing high FM Curie temperatures over 300 K.

**Figure 3.**
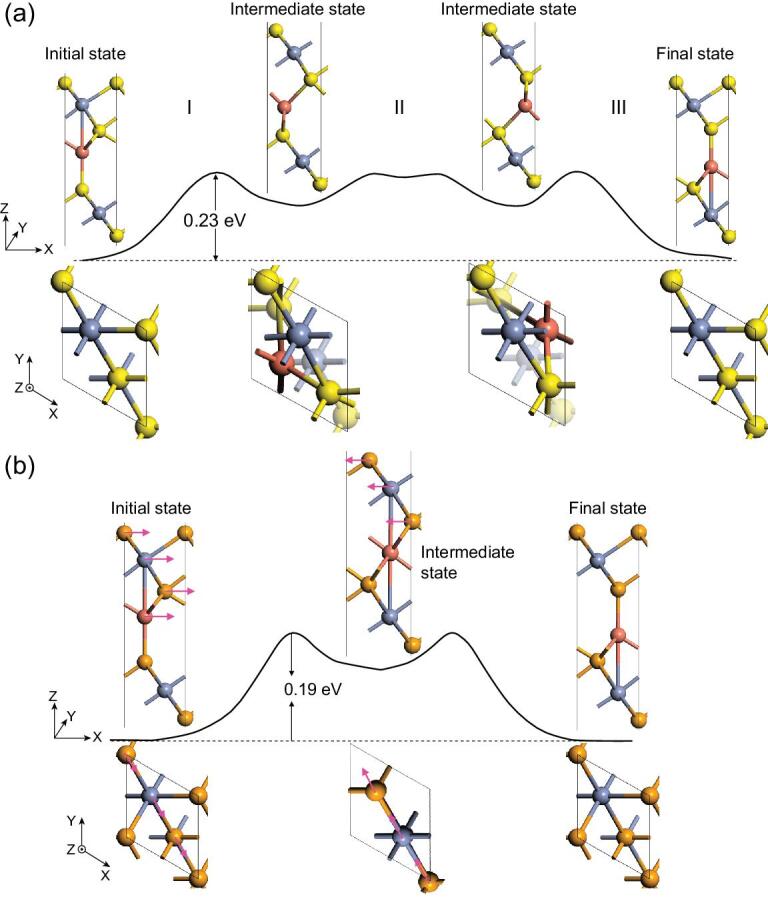
FE switching pathways by NEB method for (a) Cu(CrS_2_)_2_ and (b) Cu(CrSe_2_)_2_, where the sideview and overview of intermediate states are shown. Each Cu ion respectively binds with 4 S atoms and 6 Se atoms in the displayed intermediate states where the CrX_2_ layers are also simultaneously displaced in-plane (see pink arrows), distinct from the pathway of the bulk phase in Fig. 1.

Given the ferromagnetic ultrathin structure, it would be highly attractive to check the ferroelectric stability, considering the general belief that they should be intrinsically exclusive. Indeed, although the bilayer system is in-plane metallic as a result of enhanced carrier density, the electrons are confined vertically and the vertical polarizations will not vanish; they are both around 2.0 × 10^−12^ C/m for Cu(CrS_2_)_2_ and Cu(CrSe_2_)_2_ as listed in Table S2. Given this ferroelectric stability, one may also check the FE switching by calculating the switching pathways using the NEB method, as shown in Fig. 3, to check whether the barrier for such switching can be reduced or not. It is noted that the intermediate states are local minima without soft modes. The calculated switching barriers are respectively reduced to 0.23 eV and 0.19 eV per Cu ion compared with the bulk lattice, which might be attributed to those low-energy intermediate states with in-plane translation of CrX_2_ layers simultaneously. For this case, it is noted that the polarization can be switched if the layers are thinner than 1.0 nm where a vertical electric field of 1 V/nm could be induced by a voltage less than 1 V. Furthermore, the robustness of the FE state at ambient conditions can be confirmed by *ab initio* molecular dynamics (MD) simulations shown in Fig. S2, where the FE structure is still maintained at the end of 5 ps at 300 K. Note that the antiferroelectric configuration in Fig. S3 is highly unfavorable in energy, more than 100 meV higher compared with the FE state.

Given the prediction of coexisting ferroelectricity and ferromagnetism, one comes to discuss the possible ME coupling by checking the magnetization switching for a bilayer structure. Our calculation suggests that such a switching is not possible. However, we note that the magnetic moments of Cr_1_ and Cr_2_ in Fig. 2a are respectively 2.71 and 2.55 μ_B_, which are different due to the vertical polarization shifting the occupied t_2g_ orbital of Cr_2_ above the Fermi level, as revealed in Fig. 2a. Upon the FE switching, the magnetic moment of Cr_1_ and Cr_2_ will be swapped, giving rise to a magnetic moment transfer from the upper layer to the lower layer.

### Thickness dependence

**Figure 4.**
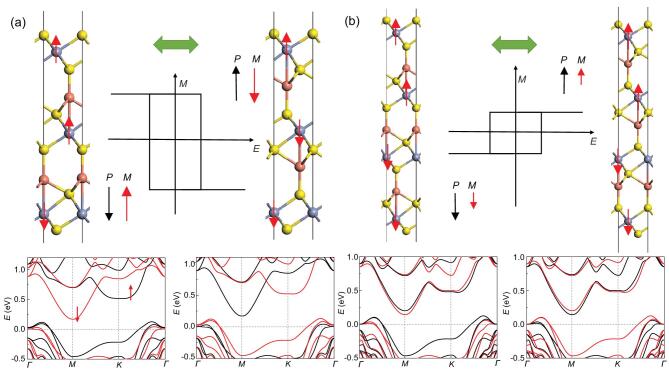
The change of spin distribution and band structure upon FE switching for (a) Cu_2_(CrS_2_)_3_ and (b) Cu_3_(CrS_2_)_4_. Black and red arrows in the sketches of *M*–*E* loops denote the directions of polarization and magnetization, respectively. The smaller net magnetization marked by the shorter red arrow in (b) is mainly attributed to the reduction in the magnetic moment of the top layer compared with other layers (left), which is ‘transferred’ to the bottom of a different spin direction after FE switching (right). Black and red lines in band structures denote spin-up and spin-down channels, respectively.

It is interesting to find that the ME coupling can be greatly enhanced in tri-layer, which will be demonstrated below. Tri-layer Cu-intercalated CrX_2_, denoted as Cu_2_(CrX_2_)_3_, is still completely FM for X = Se while Cu_2_(CrS_2_)_3_ possesses much more complex spin configurations. Although the magnetic coupling is FM in-plane for Cu_2_(CrS_2_)_3_, we still need to check various possible configurations of interlayer coupling as listed in Table. S3. The FM state is higher in energy compared with three ferrimagnetic states, and the ground state turns out to be the configuration in Fig. 4a, with a total magnetization of 2.62 μ_B_/f.u.. When the polarization is aligned downwards, the middle layer is FM coupled with the top layer while AFM coupled with the lower layer; as the polarization is reversed, according to the symmetry, the middle layer should be AFM coupled with the top layer and FM coupled with the lower layer in the ground state. The energy will be lowered by 16.4 meV with the magnetization reversal of the middle layer (see Table S3), which should take place spontaneously considering the small barrier of spin switching equivalent to the spin anisotropy energy (< 1 meV). As a result, the 180-degree reversal of a considerable magnetization of 2.62 μ_B_/f.u. can be achieved via FE switching. Similarly, four-layer Cu-intercalated CrX_2_, denoted as Cu_3_(CrX_2_)_4_, is still FM for X = Se and ferrimagnetic for X = S. The comparison of different spin configurations for Cu_3_(CrS_2_)_4_ is also displayed in Table S3. The ground state turns out to be the configuration in Fig. 4b, where the upper two layers are FM coupled, which are AFM coupled with the lower two layers. The magnetic moments of each Cr ion in the first, second, third and fourth layers are respectively 2.59, 2.80, −2.82 and −2.75 μ_B_ as the polarization points downwards, where the magnetic moment of the first layer is reduced due to the orbital shifting via vertical polarization. The total net magnetization of 0.35 μ_B_/f.u. will also be reversed upon FE switching, where the magnetic moment of each Cr ion in the first, second, third and fourth layers will be respectively changed to 2.75, 2.82, −2.80 and −2.59 μ_B_. The FE switching also gives rise to the swapping of the spin-up and spin-down channels in band structures and renders ‘electrical writing + magnetic reading’.

**Figure 5.**
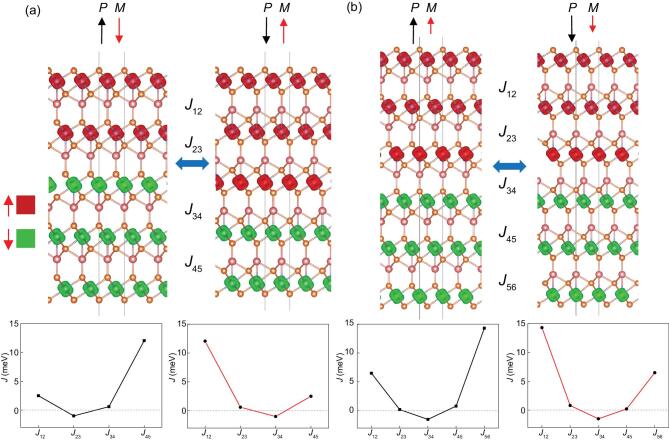
The change of spin distribution upon FE switching and interlayer coupling constant *J* for (a) Cu_4_(CrSe_2_)_5_ and (b) Cu_5_(CrSe_2_)_6_. Black and red arrows on the top denote the direction of polarization and magnetization, respectively. The smaller net magnetization marked by the shorter red arrow in (b) is mainly attributed to the reduction in the magnetic moment of the top layer compared with other layers (left), which is ‘transferred’ to the bottom of a different spin direction after FE switching (right). Red and green blocks represent isosurfaces of spin-up and spin-down density, respectively. The increase of *J* from positive to negative values reveals a transition from FM at the surfaces to AFM inside, and the asymmetrical distribution of *J* from inside to two surfaces should be attributed to the vertical polarization, which is also electrically switchable.

When the thickness increases to five layers, the Cu-intercalated CrSe_2_ denoted as Cu_4_(CrSe_2_)_5_ also turns from ferromagnetic to ferrimagnetic. Figure 5a displays the ground state of various spin configurations listed in Table S3, with a total net magnetization of 2.69 μ_B_/f.u.: when the polarization is aligned upwards, the upper two layers are FM coupled, which are AFM coupled with the three layers below; as the polarization is reversed, the magnetization of the middle layer will also be reversed, leading to a 180-degree reversal of a net magnetization of 2.69 μ_B_/f.u.. Similarly, the ground state of six-layer Cu-intercalated CrSe_2_, denoted as Cu_5_(CrSe_2_)_6_, turns out to be the configuration in Fig. 5b, where the coupling between the third and fourth layers is AFM while other couplings between adjacent layers are FM. The magnetic moment of each Cr ion from the first to the sixth layer are respectively 2.91, 2.93, 2.94, −2.94, −2.94 and −2.76 μ_B_, and the total net magnetization of 0.335 μ_B_/f.u. will also be reversed upon FE switching. The interlayer constant *J* between adjacent layers can also be calculated from the energy difference of different spin configurations in Table S3, revealing the trend of rising from negative values (AFM) inside to positive values (FM) outside. This should be plausible noting that the inside layers share similar configurations with the AFM bulk phase, while the surface layers with only one side binding with Cu ions are hole-doped in comparison so the FM coupling can be strengthened by enhanced carrier density. The energy cost for a spin to flip in the central layer will be much lower compared with in the two surface layers, which is the reason in Figs 4a and 5a why the magnetization of the central layer instead of two surface layers should be reversed, although those two final states (-P, -M) and (-P, M) will be degenerate in energy. Here the energy difference between FM and some ferrimagnetic states is within 2 meV, which may be sometimes beyond the accuracy by density-functional theory (DFT) calculations. Meanwhile the carrier density of Cu*_n_*(CrSe_2_)*_n_*_+1_ will be reduced with increasing *n*, which facilitates AFM against FM coupling. The bulk structure is AFM while the bilayer is FM, so in reality the FM to ferrimagnetic transition at some critical thickness is expected, and the AFM regions inside will be enlarged with increasing thickness while the hole-doped surface layers maintain FM.

## CONCLUSION

In summary, we show first-principles evidence of 2D room-temperature multiferroicity in ultrathin-layer CuCrS_2_ and CuCrSe_2_, where the mutual exclusions that hinder the coexistence of robust magnetism/ferroelectricity and strong magnetoelectric coupling in traditional multiferroics can be resolved. Their ferromagnetism can be stabilized due to enhanced carrier density and orbital shifting by the vertical polarization, and the Curie temperature of both ferromagnetism and ferroelectricity can be above room temperature. Layers with different thicknesses may exhibit diversified types of magnetoelectrics for efficient ‘magnetic reading + electrical writing’ due to the gradient of the interlayer coupling parameter, where a considerable net magnetization can be reversed upon FE switching. Our prediction not only exploits new types of multiferroic couplings in 2D, but also proposes a way of constructing robust multiferroics for practical applications, which may stimulate experimental efforts concerning the recent synthesis of previously predicted intrinsic 2D ferroelectrics.

## METHODS

The theoretical calculations were employed based on DFT methods implemented in the Vienna Ab initio Simulation Package (VASP 5.3.3) code [37,38]. The generalized gradient approximation (GGA) in the Perdew–Burke–Ernzerhof (PBE) [39] exchange-correlation functional and the projector augmented wave (PAW) [40] formalism were applied. We note that the PBE functional has been applied in previous studies on CuCrX_2_ and fits well with the experimental data [35]. We have checked our results by using PBE + *U* (*U* = 5 eV) and the Heyed–Scuseria–Ernzerhof (HSE) hybrid functional [41], respectively. It turns out that the ground-state spin configurations obtained by PBE and HSE are the same, which is distinct from the results obtained by PBE + *U* in some cases. The kinetic energy cut-off was set at 520 eV, and the Brillouin zone was sampled by Γ-centered 13 × 13 × 1 k points using the Monkhorst–Pack scheme [42]. The convergence threshold for self-consistent-field iteration was set to be 10^−6^ eV and the atomic positions were fully relaxed until the forces on each atoms were less than 0.001 eV/Å. A vacuum space of 17 Å was set in the vertical direction for 2D systems. The Berry phase method is employed to evaluate crystalline polarization of bulk CuCrX_2_ [43], and dipole moment correction is applied to evaluate the vertical polarizations of thin films, which can give approximately the same value experimentally measured in metallic WTe_2_ bilayer [13]. The FE switching pathway is calculated by using the climbing image nudged elastic band (NEB) method [44].

## Supplementary Material

nwz169_Supplemental_File
