# Supplementary material for: Room-temperature multiferroicity and diversified magnetoelectric couplings in 2D materials
Source: Natl Sci Rev. 2019 Nov 5;7(2):373–80. doi: 10.1093/nsr/nwz169 (PMC8288967; doi:10.1093/nsr/nwz169)
Supplement: nwz169_Supplemental_File [file nwz169_supplemental_file.docx]

Supplementary Data

Room-Temperature Multiferroicity and Diversified Magnetoelectric Couplings in Two-Dimensional Materials

Tingting Zhong^1^, Xiaoyong Li^1^, Menghao Wu^1*^, Jun-Ming Liu^2^

^1^School of Physics and Wuhan National High Magnetic Field Center, Huazhong University of Science and Technology, Wuhan, Hubei, China 430074

^2^Laboratory of Solid State Microstructures, Nanjing University, Nanjing, China 210093


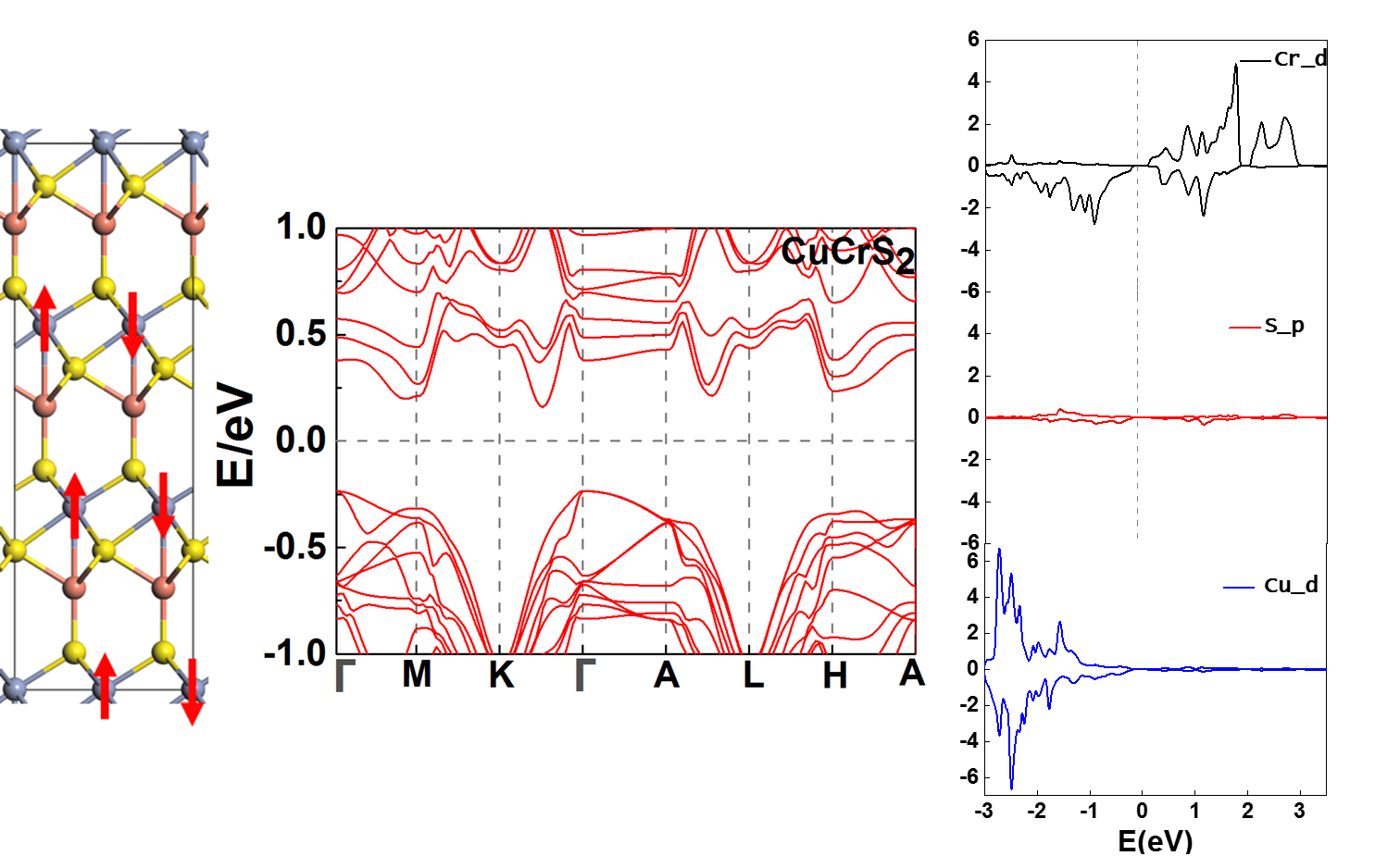


Figure S1. Ground state spin configuration, bandstructure and PDOS analysis of bulk CuCrS_2_.

|  | Cu(CrS_2_)_2_ | Cu(CrSe_2_)_2_ |
| --- | --- | --- |
| J_1_ (meV) | 23.5 | 22.9 |
| J_2_ (meV) | 2.45 | 4.32 |

Table S1. J_1_ and J_2_ respectively defined as the nearest intralayer and interlayer neighboring exchange coupling parameter, which is calculated from the energy difference between different spin configurations using HSE functional.

|  | Cu(CrS_2_)_2_ | Cu(CrSe_2_)_2_ | Cu_2_(CrS_2_)_3_ | Cu_2_(CrSe_2_)_3_ | Cu_3_(CrS_2_)_4_ | Cu_3_(CrSe_2_)_4_ |
| --- | --- | --- | --- | --- | --- | --- |
| P ($\boldsymbol{10}^{\boldsymbol{-12}}$ C/m) | 2.01 | 1.90 | 1.87 | 2.25 | 1.86 | 2.20 |


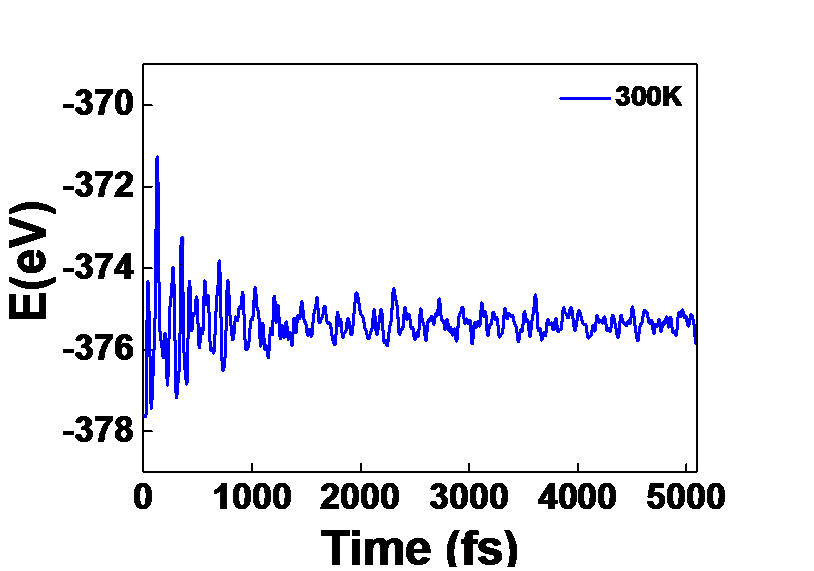
Table S2. The polarizations of Cu-intercalated thin layers.


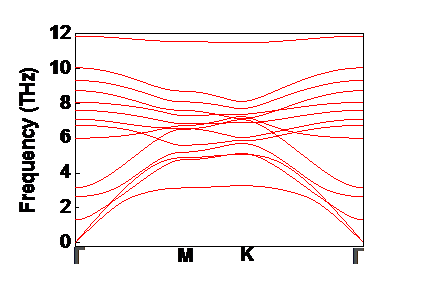


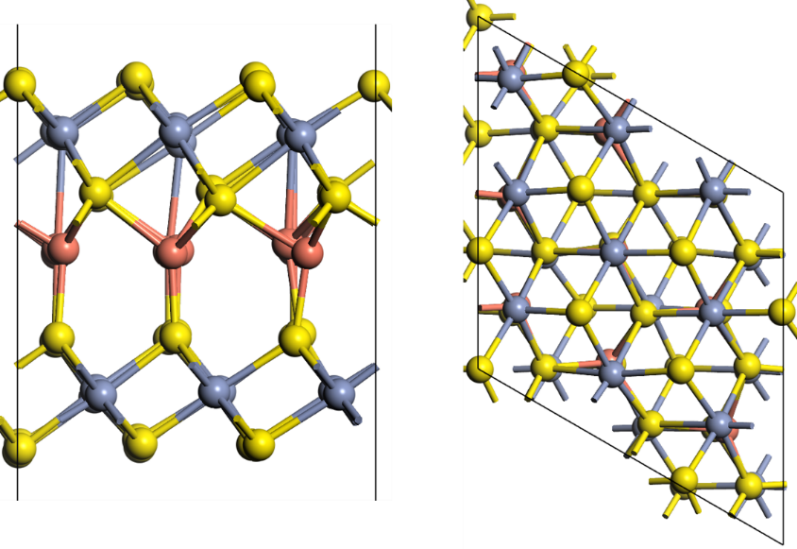


Figure S2. Snapshot of the equilibrium structures at 300K for Cu(CrS_2_)_2_  at the end of 5ps of ab initio MD simulation with a canonical ensemble (time step is set as 1fs) and the energy evolution of total energy. The phonon spectrum free of imagine frequency is also displayed.


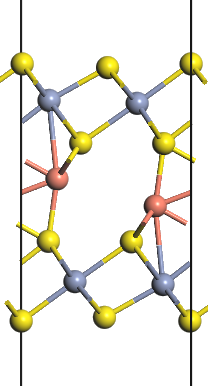


Figure S3. Antiferroelectric configuration of Cu(CrS_2_)_2_ .


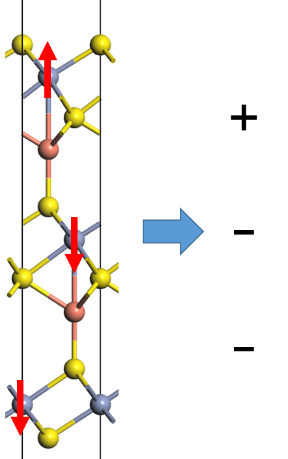


|  | Cu_2_(CrS_2_)_3_ | | | Cu_3_(CrS_2_)_4_ | | |
| --- | --- | --- | --- | --- | --- | --- |
|  | +  +  - | +  -  + | +  -  - | +  -  -  + | +  -  +  - | +  +  -  - |
| ΔE(meV/unitcell) | 16.4 | 15.7 | 0 | 22.4 | 11.9 | 0 |

|  | Cu_4_(CrSe_2_)_5_ | | |  |  |  |  |  | | |  |  |  |
| --- | --- | --- | --- | --- | --- | --- | --- | --- | --- | --- | --- | --- | --- |
|  | -  +  -  +  - | -  +  +  -  - | +  +  +  -  - | +  -  +  -  - | +  +  -  -  - | +  -  -  +  - | -  -  +  +  - | +  -  +  +  - | -  +  +  +  - | -  -  +  -  + | -  +  +  +  + | +  +  +  +  - | +  +  +  +  + |
| ΔE(meV/unitcell) | 12.6 | 3.8 | 1.2 | 2.2 | 0 | 13.8 | 12.0 | 14.0 | 14.1 | 10.3 | 13.1 | 3.5 | 1.0 |

|  | Cu_5_(CrSe_2_)_6_ | | |  |  |  |  |  | | |
| --- | --- | --- | --- | --- | --- | --- | --- | --- | --- | --- |
|  | -  +  -  +  -  + | -  +  +  -  +  - | -  +  +  -  -  + | +  +  +  +  +  - | +  +  +  +  -  - | +  +  -  -  -  - | +  -  -  -  -  - | -  -  +  +  -  - | +  +  +  -  -  - | +  +  +  +  +  + |
| ΔE(meV/unitcell) | 16.4 | 18.6 | 20.6 | 15.8 | 2.3 | 1.6 | 8.0 | 2.2 | 0 | 1.5 |

Table S3. Energy difference of various spin configurations compared with ground state, where + and – respectively denote spin-up and spin-down of each layer when the polarization is aligned upwards, as shown in the graph.
